# Beta-glucans supplementation for sows during gestation and lactation

**DOI:** 10.5713/ab.24.0516

**Published:** 2025-01-24

**Authors:** Julia Delpupo Coelho, Pedro Henrique Watanabe, Tiago Silva Andrade, Rayssa Aline Rocha Teixeira, Marcelo Emersom Costa Santos, Ingrid Barbosa de Mendonça, Deborah Marrocos Sampaio Vasconcelos, Isaac Neto Goes da Silva, Manoel Wanamark David Ferreira Filho, Leonardo Augusto Fonseca Pascoal, Ednardo Rodrigues Freitas

**Affiliations:** 1 Departamento de Zootecnia, Universidade Federal do Ceará, Fortaleza, CE, Brazil; 2 Faculdade de Veterinária, Universidade Estadual do Ceará, Fortaleza, CE, Brazil; 3 Departamento de Ciência Animal, Universidade Federal da Paraíba, Bananeiras, PB, Brazil

**Keywords:** Beta-Glucan, Colostrum, Gestation, Immunity, Lactation, Parity, Sows

## Abstract

**Objective:**

The study aimed to evaluate the effects of dietary beta-glucan supplementation for gestation and lactation sows of 1st, 2nd or 3rd parity on reproductive parameters, litter performance, milk composition and Brix value of colostrum, hemogram and serum immunoglobulin G concentration of sows and piglets.

**Methods:**

A total of 78 sows were distributed in a completely randomized design, using a 2×3 factorial scheme. This design included two supplementation levels of beta-glucans (0 and 450 mg/kg) and three parities (1st, 2nd, and 3rd), resulting in six treatments with 13 replicates each. Supplementation was administered from the beginning of gestation until the end of lactation.

**Results:**

No interaction was observed between supplementation and parity for the analyzed variables. The dietary supplementation of purified beta-glucans did not influence the reproductive performance of sows, litter performance, and the immune response of sows during gestation and lactation. The weight gain and colostrum Brix value were higher (p<0.05) in 2nd and 3rd compared with 1st parity sows.

**Conclusion:**

Dietary supplementation of beta-glucans for gestation and lactation sows of 1st, 2nd, or 3rd parity did not affect the performance of sows and litters; however, 2nd and 3rd parity sows had heavier litters, greater weight gain in the suckling period, and higher values of colostrum Brix than 1st parity sows.

## INTRODUCTION

Studies on sow’s nutrition have intensified, due to the direct relationship between the development of piglets and sow condition from the beginning of gestation to the end of lactation [1]. In this sense, promoting intestinal health in sows during reproductive development aims to improve not only newborn piglets’ performance but also prolifically and longevity during reproductive life.

Among the main compounds evaluated for pigs, prebiotic additives have been studied for their possible intestinal microbiota modulation, emphasizing beta-glucans, present in ingredients of plant origin, fungi, bacteria, and yeast. Formed by heterogeneous groups of glucose polymers linked by a linear β-glycosidic chain center 1 → 3, beta-glucan can have variable branches, the main ones being the 1 → 4 and 1 → 6 glycosidic chains [2]. Despite being found in several sources, the extraction of beta-glucans from the yeast cell wall is the most explored due to their availability as waste from the sugar-energy industry [3]. From this source, yeast cell wall beta-glucans consist of 1→3 β-linked glucopyranosyl residues with small numbers of 1→ 6 β-linked branches [4].

Beta-glucans have been researched not only for their prebiotic action but also due to their possible antioxidant, immunostimulating, and anti-inflammatory potential [5]. Due to their antioxidant and anti-inflammatory action, beta-glucans have activity in eliminating hydroxyl radicals, regardless of the extraction method and molecular size, thus also inhibiting the production of intracellular reactive oxygen species, inhibiting the production of pro-inflammatory mediators [6,7]. In addition, beta-glucans act as an immunostimulating agent, through the activation of macrophages and natural killer cell cytotoxicity [8]. Based on the bioactivity of beta-glucans, dietary supplements were developed to promote intestinal health and improving the immune status of sows and, consequently, the performance of litters [9].

Beta-glucan supplementation has demonstrated implications for the variability of the intestinal microbiota of pregnant sows [10], positively affecting the intestinal health of piglets at weaning [11]. In piglets, it has been observed that dietary supplementation of beta-glucans induces a more anti-inflammatory or tolerant state of the immune system [12]. In this sense, the same effect could be promoted in pregnant pigs that also receive many vaccines at this stage. Despite this, there is no consensus on the benefits of dietary inclusion of beta-glucans and studies present conflicting results, related to the level and period of supplementation [3,11].

The immunological transfer from gilts and sows to their litters tends to be different, considering that older sows, due to their greater exposure to infectious agents, develop robust immune responses, resulting in colostrum with higher concentrations of immunoglobulin G (IgG) and also provide passive immunity to piglets during their first few weeks of life [13]. In this sense, it was hypothesized that the dietary supplementation of beta-glucans to sows at the beginning of their reproductive life could be more beneficial, so the use of this additive could also provide a better immune response to piglets.

The objective of the present study was to evaluate the effects of dietary supplementation of beta-glucans fed to sows of 1st, 2nd, or 3rd parity during gestation and lactation on reproductive performance, productive parameters of litters, milk composition, brix value of colostrum, blood count, and serum IgG from sows and piglets.

## MATERIAL AND METHODS

The experimental protocol was approved by the Ethics Committee for the Use of Farm Animals (CEUAP-UFC) under protocol: 2406202201. The experiment has been located on a commercial farm in Caridade, Ceará, Brazil.

### Animals and experimental design

A total of 78 females from the commercial lineage (Topigs Norsvin, Curitiba, Brazil) were selected. The groups were distributed in a completely randomized design, in a 2x3 factorial scheme, with two beta-glucan supplementation levels (0 or 450 mg/kg) and 3 parities (1st, 2nd or 3rd parities), totaling 6 treatments with 13 replications each, with each sow and its litter considered as an experimental unit.

The sows were weighed and the backfat thickness (BT) was measured at point P2, 6.5 cm from the lumbar midline from the last rib, on both sides, using the ultrasound device (Lean-Meater; Renco Corporation, Golden Valley, MN, USA). The animals were then distributed between treatments and housed in individual cages in the gestation shed with a negative pressure refrigeration system (22.0±2.0°C).

### Diets and beta-glucan supplementation

The diets were formulated to meet the nutritional requirements (Table 1) of sows in the gestation, late-gestation, and lactation phases, following the Topigs Norsvin - TN70 Feed Manual [14]. Samples from each diet were collected for the analysis of crude protein, ether extract, and crude fiber. From insemination until 85 days of gestation, sows received the gestation diet. From 86 days until the moment of farrowing, late-gestation diet was provided and from farrowing to weaning, sows consumed lactation diet. During the experimental period, the sows were fed twice a day, and the amount supplied varied according to the parity and gestational period, following the genetics manual used. The 1st, 2nd, or 3rd parity sows received 2.0, 2.4, and 2.6 kg of diet from insemination until the 44th day of gestation, 2.2, 2.3 and 2.5 kg of diet from the 45th to the 84th day of gestation and 2.6, 2.7, and 2.9 kg of late-gestation diet from the 85th day of gestation until farrowing, respectively. Beta-glucan was previously mixed into the feed and fed to the sows at 07:00 h. The level of 450 mg/kg of product containing beta-glucans was established according to the manufacturer, based on previous results of the product. To supply 450 mg/kg of product containing beta-glucan, the supplementation was provided in 1.035 g sow per day at the gestation phase, considering the average daily feed consumption of 2.3 kg of diet and 4.05 g sow per day in lactation phase, considering the average daily feed consumption of 9.0 kg of diet.

### Reproductive performance, colostrum and blood sampling

At 110 days of gestation, weight and BT were measured, and the sows were transferred to the maternity shed. The maternity shed had negative pressure system (22.0±1.5°C) and the sows were housed in individual cages with farrowing cells containing feeders and drinkers for sows and piglets, and heated floor for the piglets. All births were monitored, the piglets were weighed, and total born, live, stillborn and mummified piglets were counted. At birth, the piglets were dried and the umbilical cord was tied and cut, followed by disinfection with a 10% iodine solution.

After the start of parturition, a colostrum sample was collected from six randomly selected animals per treatment, with 3 collections of 1 mL of colostrum being made from the pectoral, middle and inguinal teats to determine the Brix value using a portable digital refractometer (Milwaukee, Rocky Mount, NC, USA), to estimate IgG concentration [15]. A 50 mL sample of colostrum was collected, identified and stored in a −20°C freezer for subsequent analysis regarding composition in total solids, density, fat, protein, and lactose by ultrasound (LactoScan, Nova Zagora, Bulgaria).

The day after farrowing, 2 kg of feed was provided, with a progressive increase following the Topigs Norsvin - TN70 Feed Manual [14], until reaching 9 kg of feed on the 7th day postpartum, which was divided into 6 daily feedings. From the 7th day postpartum, feed was offered considering 2 kg of feed per sow plus 0.5 kg per lactating piglet and this proportion was maintained until weaning. Up to 24 hours postpartum, cross-fostering was carried out with piglets from sows with the same dietary treatment, to maintain 14 to 16 piglets per sow, with the piglets being weighed again. The piglets received pre-starter feed from the 14th day of life until weaning.

On the 7th day postpartum, blood was collected by puncture in the jugular vein with the aid of syringes and Vacutainer (BD, East Rutherford, NJ, USA) tubes from six sows and six piglets randomly selected per treatment. The samples intended for blood count were stored in EDTA tubes and sent for analysis in the laboratory. To determine the IgG concentration using an enzyme-linked immunosorbent assay (Pig IgG ELISA kit, ab291065; Abcam, Cambridge, UK), the samples were centrifuged at 2,300×g for 5 minutes, at room temperature, and the supernatants obtained were stored in Eppendorfs (Eppendorf Group, Hamburg, Germany) tubes at −20°C.

### Body composition and estimated milk production

Throughout the experimental period, piglets from different treatments were subjected to the same management, being counted again and weighed at weaning. After weaning (24 days of lactation), the sows were weighed, the BT was measured at point P2 and the days until the return to estrus were counted.

The loss of body tissue composition of the sows was estimated from the live weight (LW; kg) and BT (mm) according to the equations [16]: protein (kg) = 2.28(±2.22)+ 0.178(±0.017)LW−0.333(±0.067)BT and lipid (kg) = −26.4(±4.5)+0.221(±0.030)LW+1.331(±0.140)BT. Average daily milk production (MP) was estimated based on litter weight gain, number of piglets, and milk dry matter content [17]: MP (kg/day) = ([0.718×litter weight gain-4.9]× number of piglets)/0.19.

### Statistical analysis

Data were analyzed from sows and litters of 1st, 2nd and 3rd parity order, regarding beta-glucan supplementation, using the General Linear Models) procedure from the Statistical Analysis System 9.4 (SAS Inst. Inc., Cary, NC, USA). The model was:


Yijk=μY+Ti+βj+Tβij+ɛijk

where: ϒijk = value observed at the level of beta-glucan supplementation i (i = 0 or 450 mg/kg), at parity order j (j = 1st, 2nd and 3rd) and on repeat k (k = 1 to 13 for performance variables; k = 1 to 6 for colostrum composition and blood analysis); μϒ = population mean; Ti = effect of level beta-glucan supplementation i; βj = effect of parity order j; Tβij = effect of the interaction of level of beta-glucan supplementation i with parity order j; ɛijk = experimental error associated with the observed ϒijk value. The means were compared using the Tukey Test (p≤ 0.05).

## RESULTS AND DISCUSSION

No interaction between purified beta-glucan supplementation and parity on the reproductive performance of sows was observed (Table 2). There was also no significant effect of dietary beta-glucan supplementation on reproductive parameters. Considering the beginning of the reproductive life of sows, it is observed that the concern regarding body development is focused on primiparous and second-parity sows, although the largest litters are observed from the third parity onwards [18,19]. In this sense, it was hypothesized that beta-glucan supplementation could result in different effects depending on the parity order. There was a difference between the body weight of sows from different parities (p<0.001), which is related to the age and development of the animal. First-parity sows exhibited the highest percentage of weight loss during lactation (p<0.001), losing 23% of their body weight. This substantial loss can be explained by the significant decrease in BT observed in this group when compared to 2nd (17%) and 3rd (18%) parity sows.

Young sows have limited capacity for feed intake and digestion. This condition, coupled with the nutritional demands of growth and lactation, results in a negative energy balance, characterized by weight loss and decreased MP [20]. The BT of 2nd parity sows at insemination was the lowest among the categories analyzed, which is related to the wear caused by the first gestation and lactation. However, although less BT was observed until farrowing, weight loss did not interfere with the estrus weaning interval of these sows. Although Szuba-Trznadel et al [3] identified less post-lactation weight loss when supplementing sows with purified beta-glucans, including 0.05% in the late gestation, in the present study, beta-glucan supplementation throughout gestation did not result in the same effect. In general, purified beta-glucans have not demonstrated an effect on the weight of supplemented sows [3,10,11,18].

The main contribution of beta-glucan supplementation for sows would be related to the modulation of microbiota populations aiming at improving intestinal health [11] and immunodulation [9,21]. *In vitro* studies have shown that beta-glucans promoted increased activity of macrophages, neutrophils, and natural killer cells, and stimulated hematopoiesis [22,23]. Regarding humoral immunity, stimulation occurred through increased activation of B and T lymphocytes, increasing antibody secretion and inducing the expression of pro-inflammatory cytokines. In comparison to other studies that evaluated short periods of beta-glucan supplementation [12,19], the present study evaluated from sow insemination until weaning, with no effect on sow performance.

Similarly, no interaction was observed between dietary supplementation of beta-glucans and parity on the productive performance of litters (Table 3). Purified beta-glucan supplementation also did not influence the size and weight of litters, both at birth and at weaning. Beta-glucan supplementation in sows has not been shown to interfere with the quantity and weight of piglets, both at birth and at weaning [3,9,10]. Although some authors correlate the lack of effect with the late start of supplementation [10,21,24], in the present study there was no interaction between supplementation and the productive performance of sows, even with the start of dietary supplementation immediately after insemination. The lack of effect of beta-glucans dietary supplementation may be related to the extraction method and insufficient removal of protein extracted from yeast cells, reducing its efficiency [25]. Furthermore, although some studies indicate that beta-glucan supplementation could be related to better intestinal health [24], the sow’s body condition and adequate nutrition during the experimental trial may have been sufficient to not demonstrate the effects of supplementation.

Regarding parities, older sows are expected to have better productive performance. In the present study, 3rd parity sows produced larger and heavier litters (p<0.001). The reduction in the demand for resources mobilized for growth allows for better reproductive performance of the animal; from the third birth onwards, sows reach their productive peak [19].

Analysis of sow’s colostrum showed no interaction between supplementation with purified beta-glucans and parity (Table 4). Dietary supplementation of purified beta-glucans did not influence the production, composition and Brix value. However, parity affected the Brix value of colostrum (p = 0.005) in females in the first cycle, which had a lower Brix value when compared to sows in the 2nd and 3rd parities.

The colostrum Brix value is directly related to the concentration of immunoglobulin type G, the gamma globulin in the highest concentration in sow colostrum [15]. The results regarding the effect of beta-glucans on IgG expression may vary, with some studies observing an increase in gamma globulin [21,26], while other studies also found no effect of beta-glucan supplementation on the amount of gamma globulin in colostrum determined by immunoenzymatic assay [3,9]. dos Santos et al [27], although they did not observe an effect of beta-glucan supplementation in sows on IgG, noted higher concentration of IgA in both colostrum and milk, with the immunoglobulin fraction having the highest concentration and importance in protecting the mucosa against pathogenic microorganisms.

Based on Brix values, it is possible to infer that younger sows have a lower concentration of immunoglobulins in colostrum. Older animals are more adapted to the farm environment due to greater exposure to pathogens, they tend to have a higher concentration of serum immunoglobulins and, consequently, colostrum with a higher IgG content [13,27]. In this sense, although the initial hypothesis of increasing the concentration of immune cells in the colostrum and milk of sows through dietary beta-glucan supplementation could be positive for the beginning of the reproductive life of sows, it is observed that the effect of supplementation is greater in older sows [9,26], and that immunomodulation in gilts and sows up to the third parity order was not improved, as observed by dos Santos et al [27].

No interaction between dietary supplementation of purified beta-glucans and parity on the hematological parameters of sows and piglets was observed (Tables 5, 6). There was an influence of the parities on serum IgG in sows, with it being observed that third-parity sows had a higher concentration of serum IgG (p = 0.024). Despite this, no effects of parity on the concentration of IgG in piglets were observed. As in the present study, other authors found no influence on the use of purified beta-glucans in serum immunoglobulins in sows and piglets [3,9]. According to Chau et al. [9], the supply of IgG in colostrum is an adaptation to compensate for the sow’s inability to transmit antibodies and its lower expression observed from the 7th day of the piglet’s life coincides with the shutdown of globulin absorption in the neonate’s intestine.

Although Cabrera et al [28], also found no correlation between parity and serum immunoglobulin in piglets, beta-glucan supplementation in sows has resulted in an effect on litters through the modulation of the microbiota and the expression of genes in the intestine related to digestion and absorption of nutrients, as well as in the intestinal barrier of piglets [11], showing that this additive can be the target of studies to alleviate the challenges related to weaning piglets.

## Figures and Tables

**Table 1:** Composition of experimental diets for sows in the gestation, late-gestation and lactation phases

Item	Gestation	Late-gestation	Lactation
Ingredients (%)	100.00	100.00	100.00
Corn	76.23	72.42	59.63
Soybean meal (45% crude protein)	18.50	19.50	15.50
Extruded full-fat soybean	-	4.00	16.00
Sugar	-	-	5.00
Dicalcium phosphate	1.70	1.60	1.60
Limestone	0.50	0.40	0.60
Salt	0.50	0.50	0.50
L-Lysine HCl, 78.8%	0.04	0.05	0.32
L-Threonine	0.02	0.02	0.25
DL-Methionine	-	-	0.10
Fiber source^1)^	2.00	1.00	-
Vitamin and mineral premix^2)^	0.40	0.40	0.40
Mycotoxin adsorbent	0.10	0.10	0.10
Chemical composition			
ME^3)^ (kcal/kg)	3,18	3,23	3,33
Crude protein^4)^ (%)	15.02	16.64	18.63
Ether extract^4)^ (%)	3.34	4.00	5.82
Crude fiber^4)^ (%)	4.30	3.83	3.34
Calcium^3)^ (%)	0.850	0.800	0.887
Available phosphorus^3)^ (%)	0.534	0.521	0.523
Sodium^3)^ (%)	0.217	0.218	0.217
Total lysine^3)^ (%)	0.754	0.877	1.215
Total methionine+cystine^3)^ (%)	0.546	0.590	0.722
Total threonine^3)^ (%)	0.605	0.662	0.950
Total tryptophan^3)^ (%)	0.188	0.212	0.237

1) Citroflake B (Agrifirm do Brasil, Itupeva, Brazil).

2) Composition per kg of diet: Cobalt as cobaltous carbonate (400 mg), copper as copper sulfate (40 mg), Iron as iron sulfate (80 mg), iodine as calcium iodate (1 mg), manganese as manganese sulfate (35 mg), selenium as sodium selenite (0.36 mg), zinc as zinc sulfate (100 mg), vitamin A as retinyl acetate (10,000 UI), vitamin D3 as cholecalciferol (1,800 IU), vitamin E as DL-γ-tocopheryl acetate (30.5 IU), vitamin K3 as menadione nicotinamide bisulfite (2.5 mg), vitamin B1 as thiamine mononitrate (2 mg), vitamin B2 (5 mg), vitamin B6 as pyridoxine hydrochloride (3 mg), vitamin B12 (0.03 mg), niacin as nicotinamide (30 mg), pantothenic acid as D-calcium pantothenate (17 mg), folic acid (3 mg), biotin (0.40 mg), choline (800 mg), B.H.T. (48 g), 6-Phytase (500 FTU).

3) Calculated values.

4) Analyzed values.

ME, metabolizable energy; B.H.T., butylated Hydroxytoluene.

**Table 2:** Reproductive parameters of 1st, 2nd and 3rd parity sows supplemented with beta-glucans

Item	Suplementation (S)		Parity (P)			SEM	p-value		
	
	Control	Beta-glucans	1st	2nd	3rd		S	P	S×P^1)^
No. of observations	39	39	26	26	26				
Body weight (kg)									
At insemination	192.00	196.58	150.97^c^	190.49^b^	241.41^a^	1.287	0.070	<0.001	0.558
At farrowing	274.04	280.45	243.71^c^	275.32^b^	312.70^a^	1.852	0.078	<0.001	0.802
At weaning	219.98	226.21	187.15^c^	227^b^	255.11^a^	1.963	0.105	<0.001	0.611
Body weight loss (kg)	54.05	54.23	56.55^a^	48.29^b^	57.58^a^	1.632	0.955	0.035	0.898
Percent weight loss (%)	19.0	19.0	23.0^a^	17.0^b^	18.0^b^	0.536	0.625	<0.001	0.674
BT (mm)									
At insemination	14.84	14.30	15.36^a^	12.77^b^	15.58^a^	0,266	0.298	<0.001	0.951
At farrowing	18.48	18.00	19.00^a^	17.07^b^	18.70^ac^	0,337	0.467	0.032	0.435
At weaning	15.47	15.09	14.69^b^	14.01^b^	17.13^a^	0,374	0.602	0.001	0.743
Body tissue loss^2)^ (kg)									
Protein	8.43	8.52	8.63^a^	7.59^b^	9.2^a^	0,248	0.858	0.024	0.556
Fat	15.95	15.46	17.63	14.67	14.82	0,572	0.656	0.056	0.497
Weaning-estrus interval (d)	4.00	3.95	4.00	3.97	3.88	0,039	0.518	0.162	0.892

1) Interaction between dietary supplement of purified beta-glucans and parity orders.

2) The loss of body composition of sows was estimated from the live weight (LW; kg) and backfat thickness (BT; mm) according to the equations published by Dourmad et al [16]: Protein (kg) = 2.28+(0.178×LW)–(0.333×BT) and Fat (kg) = 26.4+(0.221×LW)+(1.331×BT).

a–c Means followed by different lowercase letters in the same line differ from each other by Tukey test (p<0.05).

SEM, standard error of means.

**Table 3:** Litter parameters of 1st, 2nd and 3rd parity sows supplemented with beta-glucans (24 days of lactation)

Item	Suplementation (S)		Parity (P)			SEM	p-value		
	
	Control	Beta-glucans	1st	2nd	3rd		S	P	S×P^1)^
No. of observations	39	39	26	26	26				
Litter size (n)									
Total born	16.57	16.76	15.79^b^	16.42^ab^	17.78^a^	0.259	0.693	0.005	0.146
Born alive	15.51	15.42	14.74^b^	15.16^ab^	16.50^a^	0.252	0.862	0.011	0.083
Cross-fostering	15.23	15.18	15.91^a^	14.81^b^	14.89^b^	0.037	0.478	<0.001	0.954
At weaning	14.57	14.68	15.27^a^	14.35^b^	14.25^b^	0.076	0.466	<0.001	0.364
Piglet weight (kg)									
Born alive	1.49	1.43	1.42	1.43	1.52	0.022	0.236	0.153	0.236
Cross-fostering	1.68	1.61	1.68	1.60	1.65	0.033	0.241	0.551	0.470
At weaning	6.49	6.71	6.09^b^	7.01^a^	6.85^a^	0.111	0.312	0.001	0.084
Piglet daily weight gain (kg)	0.21	0.21	0.19^b^	0.22^a^	0.22^a^	0.003	0.421	0.005	0.145
Litter weight (kg)									
At farrowing	23.07	21.98	20.91^b^	21.54^b^	24.80^a^	0.331	0.134	<0.001	0.492
Cross-fostering	25.68	24.42	26.86^a^	23.67^b^	24.62^ab^	0.485	0.182	0.022	0.445
At weaning	95.10	98.93	92.90	100.42	97.68	1.608	0.221	0.144	0.191
Litter daily weight gain (kg)	2.89	3.06	2.95	3.06	2.92	0.058	0.122	0.592	0.136

1) Interaction between dietary supplement of purified beta-glucans and parity orders.

a,b Means followed by different lowercase letters in the same line differ from each other by Tukey test (p<0.05).

SEM, standard error of means.

**Table 4:** Milk production, Brix value and colostrum composition of 1st, 2nd and 3rd parity sows supplemented with beta-glucans

Item	Suplementation (S)		Parity (P)			SEM	p-value		
	
	Control	Beta-glucans	1st	2nd	3rd		S	P	S×P^1)^
Milk production^2)^ (kg/day)	17.25	17.98	17.41	17.98	17.45	0.311	0.223	0.692	0.493
Colostrum compostion (No. of observations)	18	18	12	12	12				
Solids (%)	34.10	34.31	33.60	34.75	34.26	0.778	0.903	0.860	0.539
Fat (%)	14.57	13.63	14.19	14.56	13.55	0.343	0.223	0.546	0.960
Protein (%)	9.79	9.67	9.46	9.95	9.78	0.199	0.787	0.655	0.681
Lactose (%)	9.19	9.08	8.80	9.34	9.19	0.187	0.802	0.644	0.676
Brix value^3)^ (°Bx)	26.67	26.08	24.91^b^	26.87^a^	27.36^a^	0.505	0.361	0.005	0.197

1) Interaction between dietary supplement of purified beta-glucans and parity orders.

2) Milk production was based on litter weight gain (LWG), number of piglets and milk dry matter content (19%), according to the equation of Noblet and Etienne [17]: Milk production (kg/day) = ([0.718×LWG–4.9]×number of piglets)/0.19.

3) Brix value was used to estimate IgG concentration in sow colostrum, according to Hasan et al [15].

a,b Means followed by different lowercase letters in the same line differ from each other by Tukey test (p<0.05).

SEM, standard error of means.

**Table 5:** Hemogram, leukogram and serum immunoglobulin G concentration of 1st, 2nd and 3rd parity sows supplemented with beta-glucans

Item	Suplementation (S)		Parity (P)			SEM	p-value		
	
	Control	Beta-glucans	1st	2nd	3rd		S	P	S×P^1)^
No. of observations	18	18	12	12	12				
Hemogram									
Red cells (×10^6^/μL)	5.59	5.55	4.14	6.42	6.16	0.332	0.966	0.154	0.997
Hemoglobin (g/%)	11.24	11.27	10.88	11.73	11.53	0.198	0.643	0.489	0.756
Hematocrit (%)	36.36	36.38	35.50	37.00	36.62	0.489	0.985	0.684	0.495
MCV (μm^3^)	60.30	61.86	62.16	56.73	64.35	1.262	0.685	0.295	0.257
MCH (%)	31.25	31.60	30.53	31.67	32.08	0.177	0.527	0.076	0.810
Leukogram									
Total (μL)	15.49	14.87	16.03	14.74	14.78	0.651	0.757	0.822	0.957
Segmented neutrophils (%)	65.13	60.88	60.50	57.41	71.12	1.995	0.492	0.197	0.649
Eosinophils (%)	26.08	28.00	27.66	32.83	20.62	1.672	0.709	0.193	0.859
Basophils (%)	4.02	4.44	5.33	5.50	1.87	0.714	0.857	0.371	0.454
Lymphocytes (%)	0.16	-	-	0.25	-	0.048	0.146	0.144	0.144
Monocytes (%)	4.47	5.83	6.33	4.00	5.15	0.414	0.309	0.361	0.355
Platelets (10^3^/μL)	416.16	454.61	490.50	402.41	413.25	2.380	0.577	0.506	0.224
Proteins (g/dL)	7.53	6.88	6.70	7.38	7.55	0.118	0.096	0.146	0.337
Immunoglobulin G (mg/dL)	572.00	636.32	554.83^b^	562.08^b^	695.79^a^	1.792	0.172	0.024	0.574

1) Interaction between dietary supplement of purified beta-glucans and parity orders.

a,b Means followed by different lowercase letters in the same line differ from each other by Tukey test (p<0.05).

SEM, standard error of means; MCV, mean corpuscular volum; MCH, mean corpuscular hemoglobin.

**Table 6:** Hemogram, leukogram and serum immunoglobulin G concentration of piglets of 1st, 2nd and 3rd parity sows supplemented with beta-glucans

Item	Suplementation (S)		Parity (P)			SEM	p-value		
	
	Control	Beta-glucans	1st	2nd	3rd		S	P	S×P^1)^
No. of observations	18	18	12	12	12				
Hemogram									
Red cells (10^6^/μL)	5.29	5.07	5.34	4.86	5.33	0.093	0.344	0.188	0.627
Hemoglobin (g/%)	10.18	10.20	106.08	98.33	101.46	0.131	0.964	0.136	0.865
Hematocrit (%)	35.25	35.36	36.16	34.29	35.46	0.413	0.908	0.312	0.993
MCV (μm^3^)	64.32	66.91	67.88	63.72	65.25	0.871	0.234	0.251	0.912
MCH (%)	29.70	29.24	29.07	30.76	28.58	0.330	0.569	0.112	0.903
Leukogram									
Total (μl)	92.08	89.88	83.50	93.95	95.50	1.950	0.644	0.067	0.051
Segmented neutrophils (%)	55.00	56.73	63.58	39.08	61.93	2.445	0.776	0.004	0.307
Eosinophils (%)	1.00	-	0.91	1.41	1.30	0.135	0.084	0.829	0.642
Basophils (%)	37.72	38.34	32.75	53.75	27.60	2.388	0.913	0.004	0.435
Lymphocytes (%)	5.72	4.38	3.25	5.75	6.09	0.702	0.425	0.253	0.458
Monocytes (%)	676.88	761.22	664.16	749.00	744.48	2.249	0.238	0.489	0.057
Platelets (10^3^/μL)	56.11	53.31	54.66	52.00	57.46	1.041	0.278	0.276	0.169
Immunoglobulin G (mg/dL)	408.53	423.10	420.03	467.88	509.53	3.263	0.353	0.720	0.923

1) Interaction between dietary supplement of purified beta-glucans and parity orders.

SEM, standard error of means; MCV, mean corpuscular volum; MCH, mean corpuscular hemoglobin.
